# Vaccine Hesitancy in Saudi Arabia: A Cross-Sectional Study

**DOI:** 10.3390/tropicalmed7040060

**Published:** 2022-04-12

**Authors:** Olfat Alaamri, Ezzuddin A. Okmi, Yasser Suliman

**Affiliations:** 1 Saudi Public Health Authority, King Fahd Rd., Riyadh 13351, Saudi Arabia; otamari@cdc.gov.sa (O.A.); eaokmi@cdc.gov.sa (E.A.O.); 2 IQVIA Solutions Saudi Arabia Ltd., King Abdullah Ibn Abdul Aziz Rd., Riyadh 13214, Saudi Arabia

**Keywords:** vaccines, hesitancy, acceptance, healthcare professionals, Saudi Arabia

## Abstract

(1) Background: vaccine hesitancy can put the public’s health at risk from vaccine-preventable diseases. This study aimed to address vaccine hesitancy in Saudi Arabia and understand the problem’s magnitude and causes. (2) Methods: this was a descriptive observational study using quantitative and qualitative evaluation methods conducted in Saudi Arabia between December 2020 and February 2021. Public survey forms, exit interviews, and healthcare professional survey forms were used. (3) Results: our study involved 2030 public survey participants, 119 exit interviews of caregivers, and 500 healthcare professionals, demonstrating that vaccine hesitancy was relatively low. Ninety percent of the participants agreed that it was essential for everyone to receive the recommended vaccines with their children (*p* < 0.001), 92% believed that vaccines are safe for their children (*p* < 0.001), 91% of the participants agreed to give their new children all the recommended doses (*p* < 0.001), 86% welcomed mass/school vaccination campaigns (*p* < 0.001), and 81% were willing to pay for additional vaccines for themselves and their children (*p* < 0.001). (4) Conclusions: vaccine hesitancy is low in Saudi Arabia, and a positive attitude toward vaccination was detected among most of the participants. Vaccination decision-making is complex and includes emotional, cultural, social, spiritual, and political aspects.

## 1. Introduction

Vaccination is regarded as one of the greatest successes in public health. Vaccination programs save millions of children’s and adults’ lives every year by reducing mortality and morbidity due to the fact of various infectious diseases [1,2,3]. Vaccination programs rely on a high uptake rate to reduce the prevalence and incidence of vaccine-preventable diseases (VPDs). Additionally, high vaccination coverage rates provide indirect protection for the entire community, or herd immunity, by slowing the transmission of VPDs and reducing the risk of infection among high-risk groups and exposed individuals within the community [4].

Childhood immunization rates are high in most developed countries, suggesting that vaccination is still widely considered a public health measure in these countries [5]. But these national statistics could be hiding a large number of under-vaccinated people [6]. An association was found between the emergence of VPDs outbreaks, such as measles [7,8], poliomyelitis [9], and pertussis [10], and under-vaccinated or non-vaccinated communities in different countries [11]. Moreover, several studies showed that even vaccinated people might have serious concerns and worries about vaccination [12,13,14,15]. These concerns regarding vaccine safety and efficacy threaten the efforts exerted by vaccination programs worldwide [16,17,18,19,20]. Vaccine hesitance has recently been reported in Saudi Arabia. Alabbad et al. reported that 17% of their study population were hesitant to receive the influenza vaccine [21]. Moreover, Alsubaie et al. showed that vaccine hesitancy among Saudi parents was at 20% [22].

Vaccine hesitancy is defined as a delay in the acceptance or refusal of vaccines despite the availability of vaccination services to the public. Many factors influence vaccine hesitation such as timing, place, and type of the vaccine. Various elements, such as complacency, convenience, and confidence, also influence vaccine hesitancy development [23].

The broad view of vaccination hesitancy is challenging since it is not directly related to vaccine uptake, as vaccine-hesitant individuals may accept all the recommended vaccines on time but still have significant doubts about receiving the vaccines [5]. As a result, vaccine hesitancy might vary depending on the vaccine. In addition, one may feel hesitant about the flu vaccine but accept all other vaccines without hesitation. Therefore, recent vaccines typically increase vaccine hesitancy [24,25,26].

Different factors that influence vaccine acceptance, including vaccination decision-making, should be considered in a broader socio-cultural framework while considering numerous elements that might impact the decision-making process including previous experiences with healthcare services, family histories, emotions, and peer discussion [5,27].

Vaccine hesitancy can expose public health to risks of VPDs outbreaks that could have been prevented. There are several reasons for and manifestations of vaccine hesitancy that require better understanding to adequately address increasing concerns [5,28,29]. Hence, this cross-sectional study aimed to address vaccine hesitancy in Saudi Arabia, understand the magnitude and setting, and diagnose the root causes of the problem. In doing so, we can tailor evidence-based strategies to motivate hesitant caregivers/patients to accept vaccination.

## 2. Materials and Methods

### 2.1. Study Design and Participants

We conducted a cross-sectional study to assess vaccine hesitancy in Saudi Arabia. The ethics committee of the Institutional Review Board approved this study (Approval number: SCDC-IRB-A014-2020). The study was conducted according to the Declaration of Helsinki and all Saudi Centers for Disease Control and Prevention research policies. Moreover, before participating in the study, all subjects provided informed consent. All participants had the right to withdraw from the interview at any time.

In order to explore and quantify the determinants of vaccine hesitancy, a parallel exploratory design was adapted using several quantitative and qualitative research methods including surveys, exit interviews, and social media observation. The study was conducted from December 2020 to February 2021.

The study population included parents and caregivers with children aged 0–18 months, young females aged between 16 and 21 who were eligible for the human papillomavirus (HPV) vaccine, and adults eligible for complimentary vaccines including influenza, pneumococcal, and travelers’ vaccines. Healthcare professionals (HCPs) who recommend and administer vaccines to children, youth, and adults and regularly deal with vaccines and vaccination decisions were also included in our study. For the exit interviews, we included parents with children aged 0–18 months, following the vaccination of their children in vaccination clinics.

### 2.2. Sampling

According to Epi info Version [30], a sample size of 384 participants was estimated to be enough to detect a single proportion with a 5% margin of error and 95% confidence interval.

We used a stratified random sampling procedure to select the study population for the public survey. The population was stratified according to the geographical regions, socioeconomic classes, age groups, levels of education, gender, and nationality. A total of 2030 participants were randomly selected to fulfill these strata. Selected participants were recruited through random phone calls to respond to the survey’s questions. A purposive sampling method was applied for the recruitment of HCPs. They were recruited from the three main vaccination providers in Saudi Arabia including the facilities of the ministry of health, the private facilities, and other government healthcare facilities. Permission letters were obtained from the participating facilities. The respondents were selected based on their clinical profession, based on their contribution to vaccination services, and included 150 pediatricians, 80 general practitioners, 120 adult specialists, and 150 vaccine-room nurses. One hundred caregivers were also enrolled using a convenience sampling method from the vaccination clinics for the exit interviews.

### 2.3. Questionnaire Development

The study questionnaires were adopted from Vaccine Hesitancy Survey Questions Related to SAGE (Strategic Advisory Group of Experts Vaccine Hesitancy Matrix). Separate surveys were customized for each group: (1) caregivers of young children questionnaire; (2) youth and adults’ questionnaire; (3) healthcare workers’ questionnaires; (4) exit interview guide and checklist.

A panel of 3 experts reviewed the study questionnaires to test their clarity and objectivity and if they were suitable to achieving the aim of the study. A pilot study was carried out to test the feasibility and applicability of the data collection tool. Participants’ data in the pilot study were omitted from the actual study sample.

### 2.4. Data Collection

Data from healthcare professionals were collected through face-to-face interviews using written questionnaires. Moreover, data from the general population were collected by telephone through a computer-assisted telephone interview methodology. The phone interview included an explanation of the study background and objectives, collection of respondents’ demographics, and the response to the survey questions. The interviews were conducted immediately or rescheduled as per the respondents’ convenience. The estimated length of the interviews was 20 min. For the exit interviews, data were collected through face-to-face interviews using a written checklist. The estimated length of the interview was 10 min. Data from the social media were collected by the best-in-class AI-driven social listening technology platform, Crimson Hexagon.

### 2.5. Statistical Analysis

Data were collected, coded, revised, and entered into the Statistical Package for Social Science (R studio) version 4.1.1. Categorical data were summarized using frequency (n) and percentages (%), while continuous data were expressed as the mean and standard deviation (±SD). The quantitative data with parametric distribution were expressed using the range, and those with a nonparametric distribution were described using median and interquartile range (IQR). The Shapiro test was used to test the normality of distribution. The Chi-square test and Fischer’s exact test were used to compare independent categorical variables. Descriptive statistics were sub-grouped based on the geographies, age groups, level of education, socioeconomic classification (SEC), gender, and nationality. Exploratory factor analysis (EFA) was performed to make a covariance structure of the observed variables. Cronbach’s α was the measure of the reliability and internal consistency of the examined factors. While Χ2/dfRMSR and the Tucker–Lewis index were measurements of the goodness of fit of the model. The RMSEA was an indication of the error in the model. A *p*-value ≤ 0.05 was considered statistically significant.

## 3. Results

### 3.1. Sociodemographic Characteristics of Participants

A total of 2030 participants were eligible for the study. The demographic characteristics of the study population are summarized according to participants’ area (Table 1). Approximately 1321 (65%) of the participants were males, 1705 (84%) were married, and 1318 (65%) were Saudi. The age of the majority of participants ranged between 30 and 44 years (57%). Approximately half of the participants were highly educated, as many as 977 (48%) had a university or postgraduate degree.

### 3.2. Community Questionnaire

According to the SAGE Vaccine Hesitancy Matrix, we divided all survey questions into three categories—contextual influence, individual, and group influence—and other factors related directly to the vaccine and vaccination process-specific issues.

We assessed the contextual influences from historic, socio-cultural, environmental, institutional, economic, and political perspectives (Table 2). According to participants’ responses, the most trusted source of information about the vaccines was healthcare providers (76%). Social media reports were responsible for reconsidering vaccination among 1048 (52%) respondents (*p* = 0.023). Approximately 1302 (64%) thought that community leaders supported infants’ vaccination programs (*p* = 0.002), and 1321 (65%) of respondents reported no doubts if a celebrity was against a particular vaccine (*p* < 0.001), and approximately 1899 (93%) disagreed with people refusing vaccination for religious or cultural reasons (*p* = 0.036). Moreover, 1899 (94%) never rejected a vaccine because they thought it could contain DNA or other ingredients derived from religiously forbidden animals (*p* = 0.003). Approximately 1864 (92%) of the participants trusted the government to make decisions in their favor regarding the vaccine provided (*p* = 0.04). Finally, after evaluating the scree plot of the eigenvalue for the contextual influence questions, it identified six underlying constructs with eigenvalues of 1.0 or greater (Supplementary Materials Figure S1).

According to individual and group influences of vaccine hesitancy responses, as shown in Table 3, 1397 (69%) of the participants disagreed with the concept that their children would develop immunity by contracting the disease rather than receiving a vaccination (*p* = 0.027). approximately 1178 (58%) did not think there were other ways to prevent PVDs (*p* = 0.022). Approximately 867 (43%) of the participants were worried about the possibility that any of their childhood doses might not be safe (*p* < 0.001), and 866 (43%) were a bit concerned that these doses might not be able to prevent the disease (*p* = 0.012). However, 1817 (90%) of the participants agreed that it was essential for everyone to receive the recommended vaccines with their children (*p* < 0.001). After evaluating the scree plot of the eigenvalue for the contextual influence questions, six underlying constructs with eigenvalues of 1.0 or greater were identified (Supplementary Materials Figure S2).

Responses to questions directly related to vaccines are described in Table 4, and 1868 (92%) of participants thought that vaccines were safe (*p* < 0.001), and 400 (20%) considered new vaccines, such as HPV, to be safe (*p* < 0.001). However, for 286 (14%), it happened that they did not receive the vaccine in their healthcare centers due to the lack of healthcare personnel (*p* < 0.001). Finally, approximately 1643 (81%) expressed readiness to pay for additional vaccines for themselves and their children (*p* < 0.001). After evaluating the scree plot of the eigenvalue for the contextual influence questions, seven underlying constructs with eigenvalues of 1.0 or greater were identified (Supplementary Materials Figure S3).

Further, EFA model is described in Table 5 as a multivariate statistical technique to construct a covariance structure of the observed variables by the previous three parameters.

### 3.3. Healthcare Professional Questionnaire

This vaccine hesitancy survey included 500 HCPs (Supplementary Materials Table S1). All of them (100%) answered all the survey questions. The most common specialties of the participants were 158 (31.6%) pediatricians, 141 (28.2%) vaccine nurses, and 76 (15.2%) general practitioners. Among them, 187 (37.4%) were working at a private facility, 156 (31.2%) at public health centers, 125 (25%) at governmental hospitals, 276 (55.2%) of the participants were responsible for vaccine prescription, 120 (24.0%) for the vaccination clinic management, and 104 (20.8%) for vaccines administration. The main vaccination target groups were children (71.4%), adults 59.2%, and less commonly, adolescents (28.4%).

Of the participants, 48.6% had no information about the dropout rate from routine childhood vaccination last year, and 75.4% of the participants were aware of the national vaccination schedule for infants. Approximately 75.2% of the participants’ healthcare facilities spread awareness to the public regarding vaccination and immunization services through posters (29.4%), brochures (25.4%), awareness campaigns (9.8%), flyers (9.8%), social media (5.2%), hospital website (4.8%), and parents’ education (4.4%).

The most common concerns heard by the participants from the caregivers were getting sick following vaccination (53.2%) and pain (41.6%). Some children had incomplete or delayed vaccinations because of a lack of knowledge and awareness (47.2%) and fear of other side effects (40.4%).

### 3.4. Exit Interviews with Caregivers

The exit interview was carried out on 119 caregivers (Supplementary Table S2). All of them (100%) were getting the vaccinations in a governmental primary healthcare center. 61.3% of caregivers were aware of the vaccines received to their children. The most common vaccines were the DPT vaccine (39.5%), Poliomyelitis (28.6%), and Homophiles influenza (26.9%).

It was reported that 81.5% of the vaccinators informed participants about how their child might feel after the vaccination. The most common advice offered by the vaccinator to the caregivers was to use antipyretics (54.5%) or analgesics in cases of discomfort following vaccination (26.1%).

## 4. Discussion

There are several reasons for vaccine hesitancy worldwide, but we have argued that certain factors may be vital in this phenomenon. Here, we focused on the roles of media and communication, vaccine policies, and healthcare professionals on vaccine hesitancy in Saudi Arabia. Moreover, due to the recent release of Coronavirus Disease-2019 (COVID-19) vaccinations, this study aimed to guide future public health initiatives to increase population immunization rates as the COVID-19 vaccine acceptance rate was 64.7% in Saudi Arabia [31]. Moreover, COVID-19 vaccine hesitancy was reported among 31% of Saudi physicians [32]. COVID-19 also influenced the children’s vaccination decision-making, as 47.3% of Saudi parents reported their willingness to vaccinate their children against seasonal influenza in 2021 compared to 29.8% of parents in 2020 [33].

Our study involving 2030 participants demonstrated that vaccine hesitancy was relatively low among the Saudi Arabian population. There was a positive attitude toward vaccination among the majority of the participants, who were willing to contemplate taking the vaccines and administering them to their children as recommended by healthcare centers. In addition, the majority of the participants trusted the governmental instructions on immunizations. However, more than half of the study participants reconsidered vaccines after hearing negative news from social media.

Public health decision-makers and clinicians welcomed the introduction of new vaccines before the 21st century [34]. However, the number of novel vaccinations licensed and put on the market has widely increased in recent years [5]. As a result of the increase in the number of vaccines, people have become more concerned with certain vaccines or vaccine regimens [20,35,36,37]. Despite substantial evidence of vaccine safety and effectiveness, the media have played a role in keeping vaccination concerns existing. Several studies have shown that media controversy has a negative impact on vaccine uptake [38,39,40,41,42,43,44]. Although most people with health concerns still consult health experts, the Internet has become a crucial source of information [45]. There is a correlation between refusing vaccination and exploring vaccine information on the Internet [46,47]. Parents who rejected vaccination programs were substantially more likely to have negative thoughts regarding vaccine safety and their capacity to sustain their child’s health [48].

The majority of our study participants were males (65%), which may have resulted from the sampling methods used and might reflect that Saudi females are excluded from family decision-making. However, this situation is expected to improve by implementing the Saudi Arabian social and economic strategic program 2030 Vision.

Our study revealed that the most trusted source of information about vaccines were doctors (76%). Media was the least trusted information source about vaccines (3.7%). However, reports from social media made 52% of the respondents reconsider vaccinating their children, and 92% of the participants trusted the government to decide in their favor regarding the vaccine provided.

In many countries, there is a high level of vaccine safety surveillance. However, the general population and some healthcare providers do not fully understand how reliable these systems are. Misleading information about vaccination safety and the process leading to vaccine license and inclusion in universal programs is extensively disseminated, causing considerable challenges for public health doctors, policymakers, and patients [49,50].

Higher importance is given to the supposed risks of vaccines than the actual risks of diseases. Hence, we can consider that vaccines are victims of their success [35,51]. Several public health strategies showed no improvement in vaccine uptake rates when focusing on education and information [52]. This finding may be because many interventions were created to explain that vaccination rejection could be overcome by providing information about vaccine risks and benefits. However, most anti-vaccine groups claim that those with questions or doubts about vaccination are emotionally unstable, uninformed, or influenced by anti-vaccination organizations [5]. A binary “yes/no” approach to risk may also be more comfortable for the public than the probabilities used to define risk in science. Hence, public health authorities should move to design messages customized to the audience’s requirements, leverage new means such as social media, and be proactive instead of reactive to vaccination concerns [45,53].

Our study revealed that 69% of participants disagreed with the concept that their children would develop their immunity by contracting the disease rather than receiving a vaccination, 43% of the participants were a bit worried about the possibility that any of their childhood doses might not be safe, and 43% were a bit concerned that these doses might not be able to prevent the disease. However, 90% of the participants agreed that it was essential for everyone to receive the recommended vaccines with their children, and 81% were willing to pay for additional vaccines for themselves and their children.

The patient–provider relationship is critical to preserving vaccine confidence.

Healthcare professionals generally support vaccination. On the other hand, some of them could be classified as vaccine-hesitant. Recent research in Canada has revealed that a significant number of healthcare workers had worries about vaccination. More than one-third of respondents (37%) believed that children receive too many vaccines and that a healthy lifestyle can minimize the need for immunizations (36%) [54].

Because healthcare professionals are the most trusted source of information on vaccination for most patients, various tools and guidance have been developed to assist physicians in their talks with vaccine-hesitant or vaccine-refusing patients [55,56]. Vaccination decisions are often influenced by a lack of knowledge regarding “who, where, and when” one should be vaccinated. On the other hand, the relationship between vaccination knowledge and vaccine acceptance is unclear. Numerous studies showed that parents who chose to vaccinate their children have less information about vaccination and vaccine-preventable diseases than parents who did not. According to these studies, parents’ decisions were typically influenced by conformity rather than specific knowledge about vaccinations or vaccine-preventable diseases [57,58,59].

Recommendations from medical professionals are one of the essential factors in vaccine acceptance [24,60,61,62]. For example, considerable research in the United States found that information or reassurance from healthcare providers was reported by the majority of parents who changed their minds about postponing or not obtaining a vaccination for their child [24].

According to our vaccine hesitancy survey involving 500 healthcare professionals, 48.6% of participants did not know the dropout rate from routine childhood vaccination last year, and 75.4% of participants knew about the national vaccination schedule for infants. Before vaccinating her child, the most common words usually said to a mother were standard information about possible side effects and how to deal with them (61.4%). The most common concerns that participants heard from caregivers at the time of vaccination were getting sick after vaccination (53.2%) and pain (41.6%). Children have incomplete or delayed vaccinations because of lack of knowledge and awareness (47.2%) and fear of other side effects (40.4%). According to our survey carried out on caregivers, 61.3% of caregivers were aware of the vaccinations their children were receiving.

Studies have linked vaccination acceptability to the fact that people you respect are vaccinating themselves or vaccinating their children [63]. Based on ethnographic research carried out in six nations, people immunize their children because everyone else does [64]. According to our study, 65% of respondents had no doubts if a celebrity was against using a particular vaccine.

Refusal to get vaccinated is sometimes tied to philosophical or moral attitudes about health and immunity [5]. Many religious groups, such as Netherlands Orthodox Protestants and Amish in the United States, refuse vaccination for religious reasons [65,66]. Our study revealed that 93% of participants disagreed with people who refuse to take the vaccine for religious or cultural reasons. Moreover, 94% never rejected a vaccine if they thought it could contain DNA or other ingredients from religiously forbidden animals.

### Strengths and Limitations

This research included a large sample of population and healthcare providers, considering their vaccination perspectives and interactions with their patients.

The parallel exploratory mixed-methods study design and transdisciplinary nature of this study provide further insights into the relationships between healthcare providers’ knowledge, training, and vaccine hesitancy.

A combination of the data collected their analysis formed the necessary background of an appropriate action plan to improve vaccine communication and counseling among physicians, parents, and adolescents in Saudi Arabia.

A limitation arose from assessing vaccine discussions between health workers and caregivers through exit interviews with caregivers. Hence, the researchers did not attend the discussion in person to take notes.

The population survey was conducted in the local language, Arabic. Thus, linguistic barriers, particularly with some experts, may prove challenging for participants to respond to our interview questions.

In purposive sampling, it is difficult to defend the representativeness of the sample (i.e., the sampling achieved theoretical/analytic/logical generalization).

In telephone interviews, researchers depended on the answers provided by respondents and there was no way to validate the age group or city of respondents

The limitations of telephonic interviews with the population include lower response rates, absence of visual or nonverbal cues, and decreasing rapport.

## 5. Conclusions

Vaccine Hesitancy is relatively low among the Saudi Arabian population. Our study showed a positive attitude toward vaccination among the majority of the participants, and they were willing to contemplate taking the vaccines and administering them to their children as recommended by healthcare centers. Vaccination decision-making is complex, as demonstrated in this research. It includes emotional, cultural, social, spiritual, and political aspects and cognitive elements. Influences on vaccination hesitancy should also be considered such as the effect of public health and vaccine policy, communication and the media, and healthcare providers’ attitudes and practices. More research is needed to understand why certain health professionals and the general public still have reservations about the safety and efficacy of the vaccines.

## Figures and Tables

**Table 1:** Demographic characteristics of the study participants distributed across different regions.

Characters	Overall N = 2030	Western N = 387	Central N = 388	Eastern N = 478	Northern N = 394	Southern N = 383	*p*-Value
**Gender**							<0.001
Male	1321 (65%)	232 (60%)	296 (76%)	326 (68%)	249 (63%)	218 (57%)	
Female	709 (35%)	155 (40%)	92 (24%)	152 (32%)	145 (37%)	165 (43%)	
**Marital Status**							0.046
Single	325 (16%)	68 (18%)	77 (20%)	71 (15%)	52 (13%)	57 (15%)	
Married	1705 (84%)	319 (82%)	311 (80%)	407 (85%)	342 (87%)	326 (85%)	
**Age Category**							0.003
From 15 to 29	530 (26%)	117 (30%)	90 (23%)	104 (22%)	101 (26%)	118 (31%)	
From 30 to 44	1147 (57%)	210 (54%)	215 (56%)	298 (62%)	235 (60%)	189 (49%)	
From 45 to 59	326 (16%)	56 (15%)	71 (18%)	70 (15%)	57 (14%)	72 (19%)	
>60	27 (1%)	4 (1%)	12 (3%)	6 (1%)	1 (0%)	4 (1%)	
**Education status**							0.073
Basic education or less	149 (7%)	22 (6%)	31 (8%)	50 (10%)	23 (6%)	23 (6%)	
High school or equivalent	904 (45%)	194 (50%)	146 (38%)	218 (46%)	170 (43%)	176 (46%)	
University or postgraduate Degree	977 (48%)	171 (44%)	211 (54%)	210 (44%)	201 (51%)	184 (48%)	
**Job Status**							<0.001
Owners of large companies USD >60,000 per month	5 (0.2%)	0 (0%)	2 (0.5%)	1 (0.3%)	2 (0.3%)	0 (0%)	
Owners of medium sized companies, programmers USD 30,000–60,000 per month	46 (2%)	11 (3%)	20 (5%)	6 (1.2%)	4 (1.2%)	5 (1.2%)	
Owners of small shops/secondary school teachers USD 10,000–30,000 per month	235 (12%)	34 (9%)	65 (17%)	47 (10%)	52 (13.2%)	37 (10%)	
Paramedics, nurses, electricians, corporate employees, and primary school teachers USD 4500–10,000 per month	692 (34%)	126 (32.5%)	145 (37.3%)	151 (31.5%)	147 (37.3%)	123 (32%)	
Drivers, waiters, and factory employees USD 200–4500 per month	566 (28%)	110 (28.5%)	97 (25%)	151 (31.5%)	106 (27%)	102 (26.6%)	
Doormen, servants, and housewives USD <2000 per month	486 (24%)	106 (27%)	59 (15.2%)	122 (25.5%)	83 (21%)	116 (30.2%)	
**Nationality**							<0.001
Saudi	1318 (65%)	232 (60%)	232 (60%)	250 (52%)	291 (74%)	313 (82%)	
Resident	712 (35%)	155 (40%)	156 (40%)	228 (48%)	103 (26%)	70 (18%)	
Median (IQR) or frequency (%) Pearson’s Chi-squared test; Fisher’s exact test							

**Table 2:** Survey questions to assess contextual influences of vaccine hesitancy.

Participants Characteristics	Overall N = 2030	Western Region N = 387	Central Region N = 388	Eastern Region N = 478	Northern Region N = 394	Southern Region N = 383	*p*-Value
**A. Communication and media environment**							
**Who is the person/source you trust most in terms of obtaining information about vaccines** **?**							0.01
Doctor	1537 (76%)	290 (75%)	267 (69%)	369 (77%)	310 (79%)	301 (79%)	
Pharmacist	51 (2.5%)	10 (2.6%)	12 (3.1%)	10 (2.1%)	9 (2.3%)	10 (2.6%)	
Nurse	16 (0.8%)	1 (0.3%)	2 (0.5%)	4 (0.8%)	7 (1.8%)	2 (0.5%)	
Relatives	52 (2.6%)	11 (2.8%)	11 (2.8%)	12 (2.5%)	9 (2.3%)	9 (2.3%)	
Friends	31 (1.5%)	12 (3.1%)	8 (2.1%)	3 (0.6%)	5 (1.3%)	3 (0.8%)	
Media	76 (3.7%)	11 (2.8%)	20 (5.2%)	27 (5.6%)	9 (2.3%)	9 (2.3%)	
Others	267 (13%)	52 (13%)	68 (18%)	53 (11%)	45 (11%)	49 (13%)	
**Have the reports you’ve heard/read in the media/social media made you reconsider the choice to vaccinate your child?**							0.023
Yes	1048 (52%)	195 (50%)	195 (50%)	223 (47%)	223 (57%)	212 (55%)	
No	982 (48%)	192 (50%)	193 (50%)	255 (53%)	171 (43%)	171 (45%)	
**Do you remember the vaccine’s positive discussion debate in the media?**							0.2
Yes	816 (40%)	151 (39%)	143 (37%)	188 (39%)	179 (45%)	155 (40%)	
No	1214 (60%)	236 (61%)	245 (63%)	290 (61%)	215 (55%)	228 (60%)	
**Do you want to take this vaccine for yourself/your child?**							0.094
Yes	829 (41%)	156 (40%)	139 (36%)	196 (41%)	180 (46%)	158 (41%)	
No	1201 (59%)	231 (60%)	249 (64%)	282 (59%)	214 (54%)	225 (59%)	
**B. Influential leaders, information officials, and opposition and pro-vaccination**							
**Some groups do not agree to vaccination for different reasons.**							<0.001
Agree	586 (29%)	126 (33%)	128 (33%)	100 (21%)	116 (29%)	116 (30%)	
Disagree	1444 (71%)	261 (67%)	260 (67%)	378 (79%)	278 (71%)	267 (70%)	
**Do religious and political leaders, teachers, and healthcare workers in your community support vaccines for infants and children?**							0.002
Yes	1302 (64%)	258 (67%)	246 (63%)	335 (70%)	242 (61%)	221 (58%)	
No	728 (36%)	129 (33%)	142 (37%)	143 (30%)	152 (39%)	162 (42%)	
**Do you have any doubts if a celebrity is against a particular vaccine?**							<0.001
Yes	709 (35%)	131 (34%)	129 (33%)	138 (29%)	149 (38%)	162 (42%)	
No	1321 (65%)	256 (66%)	259 (67%)	340 (71%)	245 (62%)	221 (58%)	
**C. Religion/Culture/Sex/Socioeconomic Conditions**							
**Do you know anyone who has not taken the vaccine for religious or cultural reasons?**							0.3
Yes	127 (6.3%)	18 (4.7%)	23 (5.9%)	27 (5.6%)	28 (7.1%)	31 (8.1%)	
No	1903 (94%)	369 (95%)	365 (94%)	451 (94%)	366 (93%)	352 (92%)	
**Do you agree or disagree with these people?**							0.036
Yes	133 (6.6%)	24 (6.2%)	31 (8.0%)	20 (4.2%)	23 (5.8%)	35 (9.1%)	
No	1897 (93%)	363 (94%)	357 (92%)	458 (96%)	371 (94%)	348 (91%)	
**Have you ever rejected a vaccine because you think it contains pig DNA or other ingredients derived from forbidden animals?**							0.003
Yes	131 (6.5%)	19 (4.9%)	20 (5.2%)	22 (4.6%)	30 (7.6%)	40 (10%)	
No	1899 (94%)	368 (95%)	368 (95%)	456 (95%)	364 (92%)	343 (90%)	
**Do you refuse to take a vaccine/give it to your child if the vaccinator is male/female or has an ethnic/religious background contrary to your religion?**							0.005
Yes	266 (13%)	59 (15%)	33 (8.5%)	53 (11%)	63 (16%)	58 (15%)	
No	1764 (87%)	328 (85%)	355 (91%)	425 (89%)	331 (84%)	325 (85%)	
**D. Policy/Policy (Mandates)**							
**Do you trust your government to make decisions in your favor regarding the vaccines provided?**							0.04
Yes	1864 (92%)	355 (92%)	362 (93%)	450 (94%)	350 (89%)	347 (91%)	
No	166 (8.2%)	32 (8.3%)	26 (6.7%)	28 (5.9%)	44 (11%)	36 (9.4%)	
**Have you ever disagreed with the choice of a vaccine or a recommendation for vaccination by your government?**							<0.001
Yes	129 (6.4%)	17 (4.4%)	14 (3.6%)	28 (5.9%)	30 (7.6%)	40 (10%)	
No	1901 (94%)	370 (96%)	374 (96%)	450 (94%)	364 (92%)	343 (90%)	
**Have you ever had the impression that your government/healthcare provider has not provided you with the best vaccines available on the market?**							0.2
Yes	202 (10.0%)	37 (9.6%)	30 (7.7%)	44 (9.2%)	49 (12%)	42 (11%)	
No	1828 (90%)	350 (90%)	358 (92%)	434 (91%)	345 (88%)	341 (89%)	
**The only reason I give my children dosing is because they can go to kindergarten or school.**							0.004
Agree	1000 (49%)	209 (54%)	212 (55%)	223 (47%)	191 (48%)	165 (43%)	
Disagree	1030 (51%)	178 (46%)	176 (45%)	255 (53%)	203 (52%)	218 (57%)	
**E. Geographical Boundaries**							
**Has the distance, timing, and/or time of the clinic and/or the time needed to** **reach it or wait there and/or the costs to reach it prevented your child from being vaccinated?**							>0.9
Yes	479 (24%)	88 (23%)	94 (24%)	119 (25%)	88 (22%)	90 (23%)	
No	1551 (76%)	299 (77%)	294 (76%)	359 (75%)	306 (78%)	293 (77%)	
**If you must spend more than an hour receiving a vaccine, is it important enough to travel for it?**							0.058
Yes	1556 (77%)	288 (74%)	300 (77%)	389 (81%)	293 (74%)	286 (75%)	
No	474 (23%)	99 (26%)	88 (23%)	89 (19%)	101 (26%)	97 (25%)	
**Has your lifestyle (located in different places throughout the year) prevented you/your child from receiving a vaccine?**							0.3
Yes	418 (21%)	70 (18%)	75 (19%)	95 (20%)	89 (23%)	89 (23%)	
No	1612 (79%)	317 (82%)	313 (81%)	383 (80%)	305 (77%)	294 (77%)	
n (%)							
Median (IQR) or frequency (%)							
Pearson’s Chi-squared test; Fisher’s exact test							

**Table 3:** Survey questions to assess individual and group influences of vaccine hesitancy.

Participants Characteristics	Overall N = 2030	Western Region N = 387	Central Region N = 388	Eastern Region N = 478	Northern Region N = 394	Southern Region N = 383	*p*-Value
**A. Effects of personal perception of a vaccine or social/peer environment effects**							
**Can you tell me what the vaccine function is for the body?**							0.6
I Know	1293 (64%)	245 (63%)	256 (66%)	292 (61%)	256 (65%)	244 (64%)	
I Don’t Know	737 (36%)	142 (37%)	132 (34%)	186 (39%)	138 (35%)	139 (36%)	
**It is better for my child to develop his immunity by contracting the disease rather than receiving a dose.**							**0.027**
Agree	633 (31%)	125 (32%)	111 (29%)	127 (27%)	134 (34%)	136 (36%)	
Disagree	1397 (69%)	262 (68%)	277 (71%)	351 (73%)	260 (66%)	247 (64%)	
**Do you think there are other better ways to prevent vaccine-preventable diseases?**							**0.022**
Yes	852 (42%)	169 (44%)	153 (39%)	176 (37%)	185 (47%)	169 (44%)	
No	1178 (58%)	218 (56%)	235 (61%)	302 (63%)	209 (53%)	214 (56%)	
**Do you think babies should get dosing at a very young age?**							0.7
Yes	869 (43%)	174 (45%)	157 (40%)	207 (43%)	164 (42%)	167 (44%)	
No	1161 (57%)	213 (55%)	231 (60%)	271 (57%)	230 (58%)	216 (56%)	
**B. Knowledge/Awareness**							
**Do you know which vaccines you should take as well as your children?**							0.2
Yes	1584 (78%)	291 (75%)	299 (77%)	375 (78%)	323 (82%)	296 (77%)	
No	446 (22%)	96 (25%)	89 (23%)	103 (22%)	71 (18%)	87 (23%)	
**Do health professional/health workers provide all the information needed to respond to inquiries about vaccination?**							0.4
Yes	1686 (83%)	311 (80%)	327 (84%)	405 (85%)	331 (84%)	312 (81%)	
No	344 (17%)	76 (20%)	61 (16%)	73 (15%)	63 (16%)	71 (19%)	
**Do you trust the information you receive about vaccination/dosing?**							0.6
Yes	1814 (89%)	342 (88%)	343 (88%)	429 (90%)	350 (89%)	350 (91%)	
No	216 (11%)	45 (12%)	45 (12%)	49 (10%)	44 (11%)	33 (8.6%)	
**C. Risks/benefits (tangible, evidentiary)**							
**How worried are you about the possibility that any of your childhood doses may not be safe?**							<0.001
Very Worried	256 (13%)	53 (14%)	33 (8.5%)	48 (10%)	52 (13%)	70 (18%)	
A Bit Worried	867 (43%)	146 (38%)	173 (45%)	202 (42%)	177 (45%)	169 (44%)	
Not Worried	657 (32%)	139 (36%)	112 (29%)	179 (37%)	120 (30%)	107 (28%)	
Not Worried at All	250 (12%)	49 (13%)	70 (18%)	49 (10%)	45 (11%)	37 (9.7%)	
**Do you think vaccines are still needed even if the disease no longer prevails?**							0.8
Yes	1340 (66%)	255 (66%)	250 (64%)	325 (68%)	255 (65%)	255 (67%)	
No	690 (34%)	132 (34%)	138 (36%)	153 (32%)	139 (35%)	128 (33%)	
**How worried are you that your child may have a serious side effect** **from taking a dose?**							0.7
Very Worried	605 (30%)	107 (28%)	117 (30%)	134 (28%)	124 (31%)	123 (32%)	
A Bit Worried	794 (39%)	154 (40%)	156 (40%)	180 (38%)	150 (38%)	154 (40%)	
Not Worried	503 (25%)	102 (26%)	87 (22%)	135 (28%)	97 (25%)	82 (21%)	
Not Worried at All	128 (6.3%)	24 (6.2%)	28 (7.2%)	29 (6.1%)	23 (5.8%)	24 (6.3%)	
**How worried are you that a dose may not be able to prevent the disease?**							0.012
Very Worried	374 (18%)	68 (18%)	66 (17%)	88 (18%)	68 (17%)	84 (22%)	
A Bit Worried	866 (43%)	187 (48%)	175 (45%)	184 (38%)	162 (41%)	158 (41%)	
Not Worried	660 (33%)	113 (29%)	112 (29%)	180 (38%)	142 (36%)	113 (30%)	
Not Worried at All	130 (6.4%)	19 (4.9%)	35 (9.0%)	26 (5.4%)	22 (5.6%)	28 (7.3%)	
**D. Vaccination as a social custom compared to not needing it/harm**							
**I agree that it is important for everyone that they and their children receive the recommended vaccines.**							<0.001
Agree	1817 (90%)	350 (90%)	362 (93%)	440 (92%)	336 (85%)	329 (86%)	
Disagree	213 (10%)	37 (9.6%)	26 (6.7%)	38 (7.9%)	58 (15%)	54 (14%)	
**Have mothers/fathers in your community/circle of friends vaccinated their children with all the recommended vaccines?**							0.2
Yes	1747 (86%)	331 (86%)	342 (88%)	419 (88%)	338 (86%)	317 (83%)	
No	283 (14%)	56 (14%)	46 (12%)	59 (12%)	56 (14%)	66 (17%)	
Have you vaccinated your child?							0.15
Yes	1671 (82%)	309 (80%)	313 (81%)	411 (86%)	324 (82%)	314 (82%)	
No	359 (18%)	78 (20%)	75 (19%)	67 (14%)	70 (18%)	69 (18%)	
**Do you think that if you vaccinate your child, you protect others as well?**							0.5
**Yes**	1753 (86%)	330 (85%)	338 (87%)	417 (87%)	346 (88%)	322 (84%)	
**No**	277 (14%)	57 (15%)	50 (13%)	61 (13%)	48 (12%)	61 (16%)	
**Are you concerned that some mothers in your community are postponing or rejecting vaccines, putting your baby at risk of developing these diseases, such as whooping cough?**							0.8
Yes	1713 (84%)	328 (85%)	329 (85%)	407 (85%)	325 (82%)	324 (85%)	
No	317 (16%)	59 (15%)	59 (15%)	71 (15%)	69 (18%)	59 (15%)	
n (%)							
**Median (IQR) or frequency (%)**							
**Pearson’s Chi-squared test; Fisher’s exact test**							

**Table 4:** Survey questions to assess vaccine/vaccination specific issues of vaccine hesitancy.

Participants Characteristics	Overall N = 2030	Western Region N = 387	Central Region N = 388	Eastern Region N = 478	Northern Region N = 394	Southern Region N = 383	*p*-Value
**A. Risks/benefits (scientific evidence)**							
**Do you think vaccines are safe for you and your children?**							<0.001
**Yes, true**	1868 (92%)	357 (92%)	373 (96%)	446 (93%)	349 (89%)	343 (90%)	
**No, not at all**	162 (8.0%)	30 (7.8%)	15 (3.9%)	32 (6.7%)	45 (11%)	40 (10%)	
**Have you ever refused to vaccinate your child?**							0.5
**Yes, true**	80 (3.9%)	13 (3.4%)	10 (2.6%)	22 (4.6%)	19 (4.8%)	16 (4.2%)	
**No, not at all**	1950 (96%)	374 (97%)	378 (97%)	456 (95%)	375 (95%)	367 (96%)	
**Has your child been exposed to pain or adverse reactions after vaccination? (Adverse events following the Vaccination)**							0.9
**Yes, true**	307 (15%)	56 (14%)	57 (15%)	70 (15%)	66 (17%)	58 (15%)	
**No, not at all**	1723 (85%)	331 (86%)	331 (85%)	408 (85%)	328 (83%)	325 (85%)	
**Do you know anyone who has had a bad reaction after receiving a dose?**							0.4
**Yes, true**	240 (12%)	43 (11%)	50 (13%)	46 (9.6%)	48 (12%)	53 (14%)	
**No, not at all**	1790 (88%)	344 (89%)	338 (87%)	432 (90%)	346 (88%)	330 (86%)	
**Does your fear of being exposed to your child’s pain or fear of needles when taking the vaccine make you reluctant to get vaccinated?**							0.065
**Yes, true**	340 (17%)	56 (14%)	58 (15%)	75 (16%)	69 (18%)	82 (21%)	
**No, not at all**	1690 (83%)	331 (86%)	330 (85%)	403 (84%)	325 (82%)	301 (79%)	
**Do you trust your healthcare worker in terms of giving/giving your child the vaccine safely?**							0.2
**Yes, true**	1893 (93%)	358 (93%)	363 (94%)	456 (95%)	363 (92%)	353 (92%)	
**No, not at all**	137 (6.7%)	29 (7.5%)	25 (6.4%)	22 (4.6%)	31 (7.9%)	30 (7.8%)	
**B. The development of a new vaccine or preparation**							
**Do you consider a new vaccine such as the HPV vaccine “cervical cancer” to be safe**							<0.001
**Yes, true**	400 (20%)	71 (18%)	76 (20%)	55 (12%)	91 (23%)	107 (28%)	
**No, not at all**	121 (6.0%)	23 (5.9%)	27 (7.0%)	17 (3.6%)	26 (6.6%)	28 (7.3%)	
**I do not know**	1509 (74%)	293 (76%)	285 (73%)	406 (85%)	277 (70%)	248 (65%)	
**Do you think a vaccine is needed to prevent these diseases?**							>0.9
**Yes, true**	1781 (88%)	338 (87%)	346 (89%)	419 (88%)	344 (87%)	334 (87%)	
**No, not at all**	249 (12%)	49 (13%)	42 (11%)	59 (12%)	50 (13%)	49 (13%)	
**C. Design of the vaccination program/presentation method**							
**Do your welcome mass/school vaccination campaigns?**							<0.001
**Yes, true**	1746 (86%)	330 (85%)	356 (92%)	418 (87%)	330 (84%)	312 (81%)	
**No, not at all**	284 (14%)	57 (15%)	32 (8.2%)	60 (13%)	64 (16%)	71 (19%)	
**Do you let your child get vaccinated through the school vaccination program?**							>0.9
**Yes, true**	1713 (84%)	328 (85%)	329 (85%)	405 (85%)	331 (84%)	320 (84%)	
**No, not at all**	317 (16%)	59 (15%)	59 (15%)	73 (15%)	63 (16%)	63 (16%)	
**Have you ever refrained from getting your child vaccinated during a group vaccination campaign?**							0.078
**Yes, true**	164 (8.1%)	25 (6.5%)	26 (6.7%)	33 (6.9%)	41 (10%)	39 (10%)	
**No, not at all**	1866 (92%)	362 (94%)	362 (93%)	445 (93%)	353 (90%)	344 (90%)	
**Are you keen to vaccinate yourself/your family with the annual seasonal flu vaccination?**							0.3
**Yes, true**	1465 (72%)	286 (74%)	267 (69%)	340 (71%)	296 (75%)	276 (72%)	
**No, not at all**	565 (28%)	101 (26%)	121 (31%)	138 (29%)	98 (25%)	107 (28%)	
**D. Reliability and/or vaccine supply**							
**Has the health center/doctor’s clinic ever sent you home due to the lack of vaccine?**							0.6
**Yes, true**	352 (17%)	60 (16%)	75 (19%)	87 (18%)	67 (17%)	63 (16%)	
**No, not at all**	1678 (83%)	327 (84%)	313 (81%)	391 (82%)	327 (83%)	320 (84%)	
**At your health center, have you ever not received the vaccine due to lack of healthcare personnel?**							<0.001
**Yes, true**	286 (14%)	51 (13%)	50 (13%)	98 (21%)	44 (11%)	43 (11%)	
**No, not at all**	1744 (86%)	336 (87%)	338 (87%)	380 (79%)	350 (89%)	340 (89%)	
**E. Vaccination schedule**							
**If you have another child today, do you want him to get all the recommended doses?**							<0.001
**Yes, true**	1857 (91%)	351 (91%)	371 (96%)	445 (93%)	354 (90%)	336 (88%)	
**No, not at all**	173 (8.5%)	36 (9.3%)	17 (4.4%)	33 (6.9%)	40 (10%)	47 (12%)	
**It’s best for your kids to get fewer vaccines at a time.**							>0.9
**Agree**	1280 (63%)	246 (64%)	248 (64%)	294 (62%)	247 (63%)	245 (64%)	
**Disagree**	750 (37%)	141 (36%)	140 (36%)	184 (38%)	147 (37%)	138 (36%)	
**F. Costs**							
**Are you willing to pay for additional vaccines for yourself or your child?**							<0.001
**Yes, true**	1643 (81%)	314 (81%)	326 (84%)	406 (85%)	317 (80%)	280 (73%)	
**No, not at all**	387 (19%)	73 (19%)	62 (16%)	72 (15%)	77 (20%)	103 (27%)	
**G. The role of healthcare professionals**							
**Have healthcare professionals ever treated you with disrespect (for example, regarding your appearance, education, or cultural background) making you reluctant to return to the healthcare?**							0.2
**Yes, true**	131 (6.5%)	20 (5.2%)	33 (8.5%)	34 (7.1%)	19 (4.8%)	25 (6.5%)	
**No, not at all**	1899 (94%)	367 (95%)	355 (91%)	444 (93%)	375 (95%)	358 (93%)	
**Have you ever refused/hesitated your doctor to give you a vaccine you wanted to get for yourself/your child?**							0.3
**Yes, true**	18 (0.9%)	4 (1.0%)	4 (1.0%)	7 (1.5%)	1 (0.3%)	2 (0.5%)	
**No, not at all**	2012 (99%)	383 (99%)	384 (99%)	471 (99%)	393 (100%)	381 (99%)	
**n (%)**							
**Median (IQR) or frequency (%)**							
**Pearson’s Chi-squared test; Fisher’s exact test**							

**Table 5:** Exploratory factor analysis model fit for the three models.

Models	Cronbach’s α	Χ2/df	RMSR	Tucker Lewis Index	RMSEA	Correlation of Regression Scores with Factors
Contextual influences	0.43	19.72	0.08	0.27	0.082	0.77
Individual and group influences of vaccine hesitancy	0.61	35.5	0.09	0.46	0.096	0.86
Vaccine/vaccination specific issues	0.47	23.95	0.08	0.44	0.08	0.85

## Data Availability

Data can be available upon request from the first and corresponding author.

## Supplementary Materials

The following supporting information can be downloaded at: https://www.mdpi.com/article/10.3390/tropicalmed7040060/s1, Figure S1: Contextual influence components scree plot; Figure S2: Individual and group influences components scree plot; Figure S3: Vaccine/vaccination specific issues components scree plot; Table S1: Healthcare professional questionnaire; Table S2: Exit interviews with caregivers.
