# Supplementary material for: Vaccine Hesitancy in Saudi Arabia: A Cross-Sectional Study
Source: Trop Med Infect Dis. 2022 Apr 12;7(4):60. doi: 10.3390/tropicalmed7040060 (PMC9025486; doi:10.3390/tropicalmed7040060)
Supplement: Supplementary file 1 [file tropicalmed-07-00060-s001.zip › supplementary files.pdf]

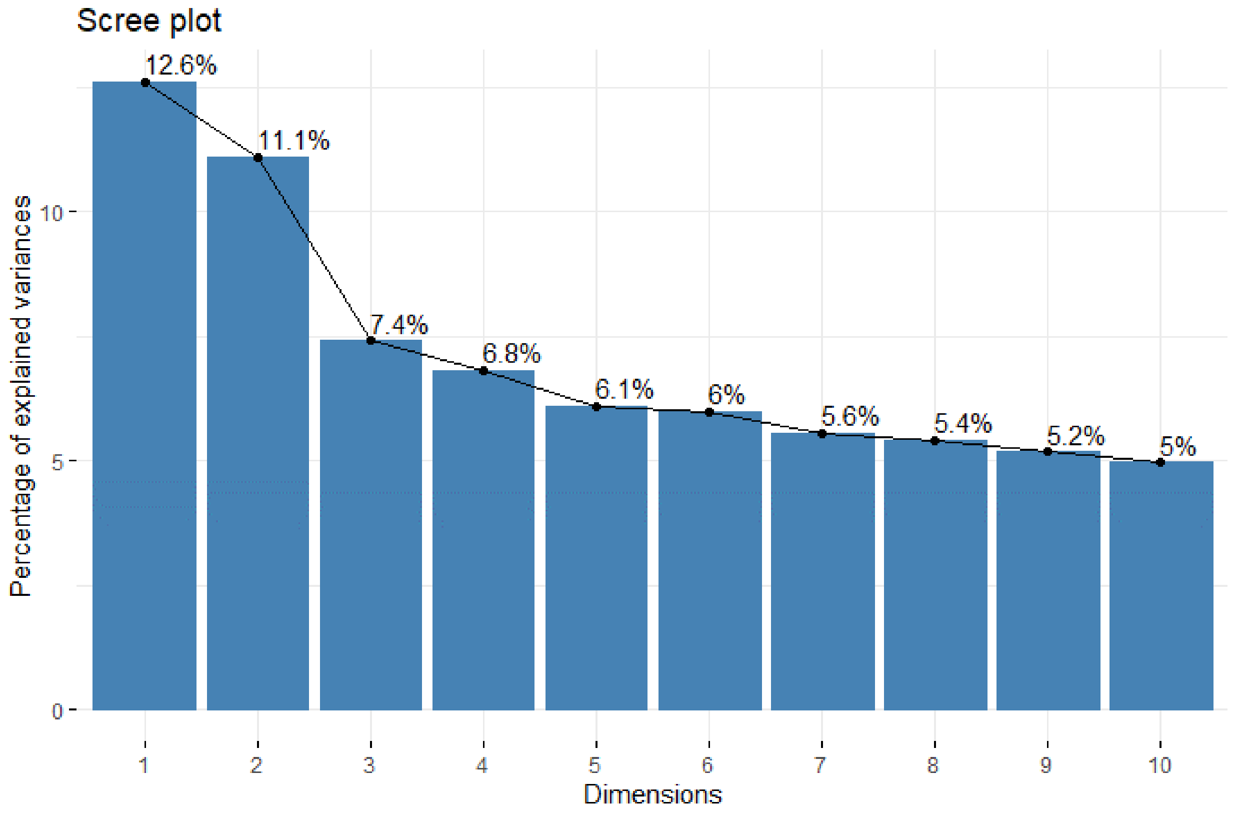

Figure S1. Contextual Influence Components Scree Plot.

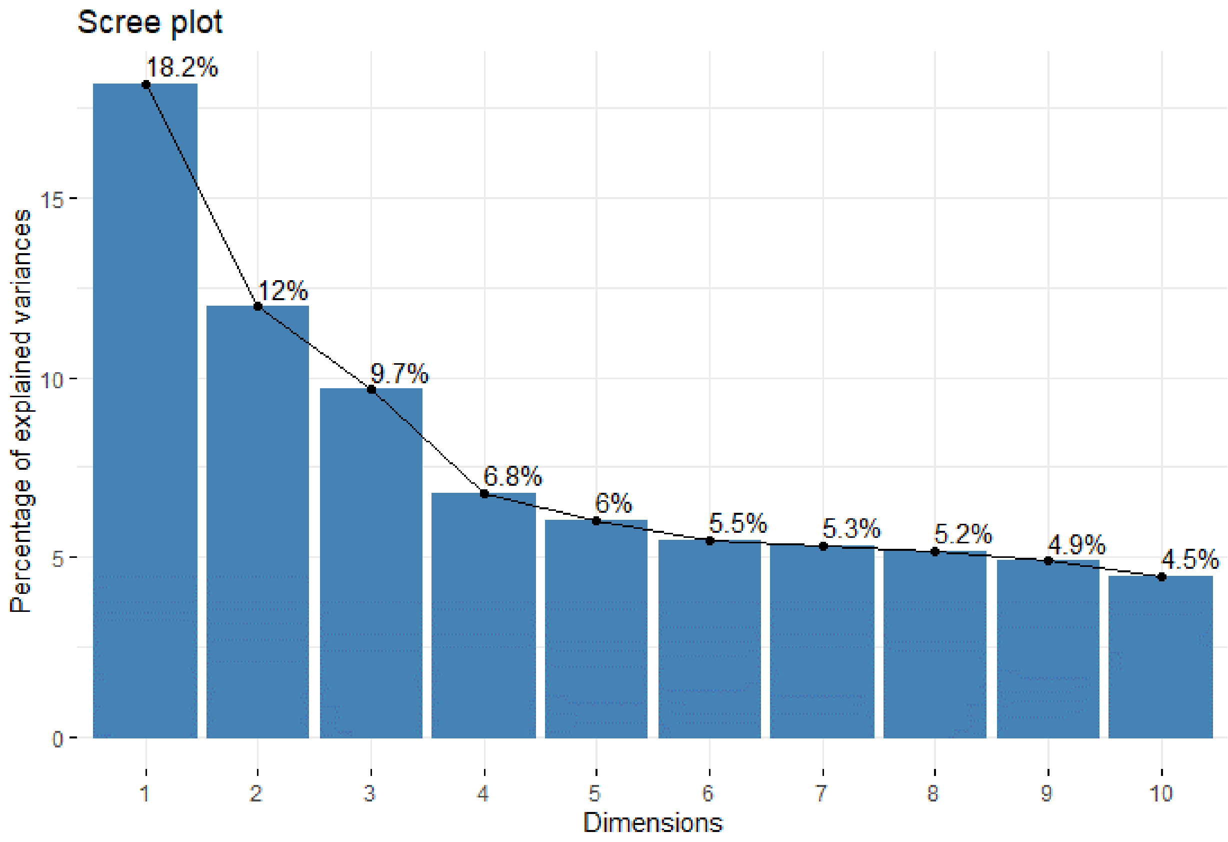

Figure S2. Individual and group influences Components Scree Plot.

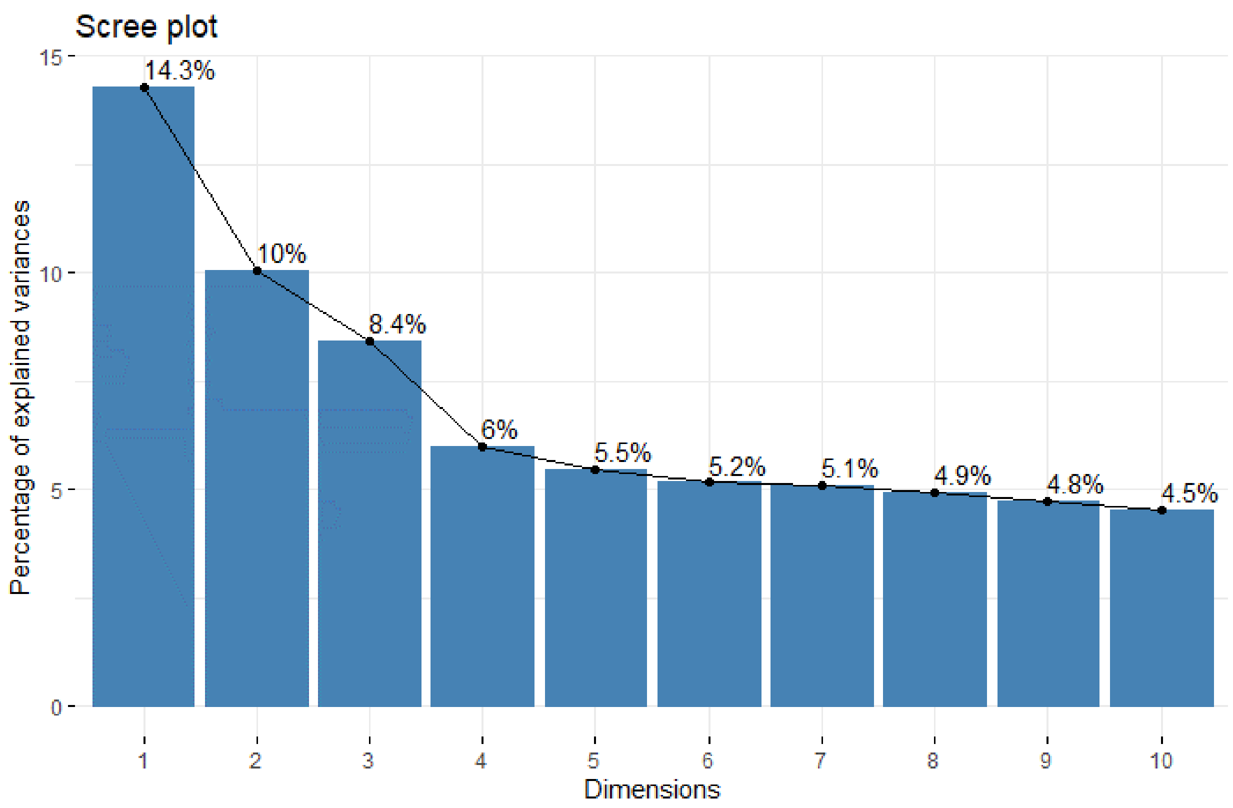

Figure S3. Vaccine/vaccination specific issues Components Scree Plot.

Table S1. Health care professional questionnaire.

**SPECIALITY:**	
Endocrinologist	40 (8.0%)
Family medicine	3 (0.6%)
General practitioner	76 (15.2%)
Gynecologist	40 (8.0%)
Pediatrician	158 (31.6%)
Pulmonologist	42 (8.4%)
Vaccine nurse	141 (28.2%)
**Type of vaccination site:**	

MOH Hospital	32 (6.4%)
Other Government hospital	125 (25.0%)
PHC	156 (31.2%)
Private facility	187 (37.4%)
**Position of person interviewed:**	
Adult specialist	122 (24.4%)
General Practitioner	78 (15.6%)
Pediatrician	150 (30.0%)
Vaccine nurse	150 (30.0%)
**Fulfilling this position for how long**	
Min.-Max.	2.0- 39.0
Mean (SD)	15.4 (7.3)
**Responsibilities related to vaccination**	
I administer vaccines	104 (20.8%)
I manage the vaccination clinic	120 (24.0%)
I recommend / prescribe vaccines	276 (55.2%)
**Main target groups**	
Children	357 (71.4%)
Adolescents	142 (28.4%)
Adults	296 (59.2%)
**Number of those who receive vaccines monthly**	
children(Min.-Max.)	0 – 2000
Mean (SD)	222.4 (354.8)
Adolescents (Min.-Max).	0 – 400

Mean (SD)	12.8 (41.9)
Adults (Min.-Max).	0 – 1500
Mean (SD)	55.3 (171.4)
**Facility's vaccination coverage for vaccines**	
All routine vaccines	357 (71.4%)
**All routine vaccines except**	15 (3.0%)
HPV vaccine	180 (36.0%)
Influenza vaccine	329 (65.8%)
Typhoid vaccine	162 (32.4%)
Hepatitis A vaccine for adults	204 (40.8%)
Meningococcal vaccine	302 (60.4%)
Varicella Zoster Vaccine	217 (43.4%)
Tetanus Vaccine	248 (49.6%)
Rabies vaccine	183 (36.6%)
Polyvalent pneumococcal vaccine (Pneumo23)	202 (40.4%)
**All routine vaccines except: (n=15)**	
BCG	8 (1.6%)
DTaP	1 (0.2%)
Hepatitis A, MMR	2 (0.4%)
HPV vaccine	2 (0.4%)
Rota and meningococcal	2 (0.4%)
**Dropout rate from children vaccination last year**	
Dropout rate	257 (51.4%)
Does not know	243 (48.6%)
Min.-Max.	0-245

Mean (SD)	17.7 (17.4)
**Are you aware of the national vaccination schedule for infants?**	
Does not know	123 (24.6%)
Knows	377 (75.4%)
**Availability of vaccines daily**	
All vaccines are available all the days	322 (64.4%)
All vaccines are available in specific days	101 (20.2%)
Not all vaccines are available	24 (4.8%)
**Availability of cold chain capacities**	
No	18 (3.6%)
Yes	482 (96.4%)
**Availability of enough staff for vaccination**	
Don't know	62 (12.4%)
No	65 (13.0%)
Yes	373 (74.6%)
**Outreach sessions, e.g., school campaigns and home campaigns**	
No	330 (66.0%)
Yes	170 (34.0%)
**Are all planned vaccination sessions outside the facility held as planned?**	
No	7 (1.4%)
Yes	163 (32.6%)
**Estimated percentage of vaccinations given outside the facility**	

Don't know	335 (67.0%)
Percentage	165 (33.0%)
Mean (SD)	24.3 (21.6)
**Feeling of most parents about vaccination**	
Accept all	352 (70.4%)
Accept but unsure / concerned	72 (14.4%)
Don’t know	70 (14.0%)
Refuse / delay some	6 (1.2%)
**Facility provide information on vaccines to the public**	
No	124 (24.8%)
Yes	376 (75.2%)
**How?**	
Posters	147 (29.4%)
Brochures	127 (25.4%)
Awareness campaigns	49 (9.8%)
Flyers	49 (9.8%)
Social media	26 (5.2%)
Hospital website	24 (4.8%)
Parents education	22 (4.4%)
Health applications	17 (3.4%)
Videos	17 (3.4%)
Leaflets and booklets	14 (2.8%)
Mobile messages	11 (2.2%)
Screens and TV advertisements	9 (1.8%)
Cards, newspaper, panniers	6 (1.2%)

**The presence of community leaders and groups in promoting immunization:**	
No	369 (73.8%)
Yes	131 (26.2%)
**The presence of a system for identifying specific infants who are behind in their vaccinations:**	
Don't know	102 (20.4%)
No	232 (46.4%)
Yes	166 (33.2%)
**What proportion of time would you estimate you spend on vaccination?**	
Min. – Max.	1-100
Mean (SD)	34.4 (34.1)
**How important is your work in vaccination?**	
Extremely important	323 (64.6%)
Somewhat important	18 (3.6%)
Very important	159 (31.8%)
**What do you normally say to a mother before vaccinating her child:**	
Number and type of vaccine/s the child will receive	295 (59.0%)
Information about the vaccine/s	289 (57.8%)
Information about the disease/s	200 (40.0%)
Information about possible side effects and how to deal with	307 (61.4%)
Nothing	10 (2.0%)
Other (n=31)	31 (6.2%)
Allergy or diseases against any medicine	2 (0.4%)
Information about next visit	2 (0.4%)
Answering their questions	2 (0.4%)
Appointment of vaccine	8 (1.6%)

Asking child history if he has disease	7 (1.4%)
period of vaccines	2 (0.4%)
Taking family history	11 (2.2%)
**What do you normally say to a mother after vaccinating her child:**	
Information about possible side effects and how to deal with	308 (61.6%)
Information about the next visit	310 (62.0%)
Nothing	37 (7.4%)
Other (n=8)	8 (1.6%)
Benefits of vaccine	4 (0.8%)
Giving full information about vaccine	2 (0.4%)
How he can eat, drink, how to deal with medications	2 (0.4%)
Make some medication if there are bad things happens	1 (0.2%)
Side effects	1 (0.2%)
**Any factors that make your conversations with caregivers at the time of vaccination difficult:**	
No	414 (82.8%)
Yes	86 (17.2%)
**Please tell me about these factors:**	
Limited time available	52 (10.4%)
There are too many children to deal with	37 (7.4%)
Limited knowledge about vaccines and related diseases	13 (2.6%)
Lack of training / orientation	10 (2.0%)
Other	11 (2.2%)
Language barriers	8 (1.6%)
Languages barriers for Expats	1 (0.2%)
**Discussing immunization and vaccination services in communities:**	

No	279 (55.8%)
Yes	221 (44.2%)
**Concerns from caregivers about vaccinations**	
Concerns about pain	208 (41.6%)
Concerns about other effects	431 (86.2%)
Concerns about infection	110 (22.0%)
Concerns about getting sick after vaccination	266 (53.2%)
Other	13 (2.6%)
**Other (n=13)**	
Doubts about vaccination benefits	5 (1.0%)
Connecting some disease with vaccine	3 (0.6%)
Fear of vaccination	2 (0.4%)
Allergy	1 (0.2%)
Useless to give vaccine	1 (0.2%)
Vaccines that might may affect pregnancy	1 (0.2%)
**How do you normally respond to these concerns about pain?**	
Explore their concern and then respond	289 (57.8%)
Provide information about vaccines	291 (58.2%)
Provide information about possible side effects and how to deal with them	406 (81.2%)
Show empathy and reassure the parent/caregiver	208 (41.6%)
Other	14 (2.8%)
Recommended some medication	12 (2.4%)
Talk about the importance of vaccine	1 (0.2%)
Announcing the importance of vaccine	1 (0.2%)

What are the main reasons why some children in your area have incomplete or delayed vaccinations?	
Fear of pain	62 (12.4%)
Fear of other side effects	202 (40.4%)
Fear of infection	49 (9.8%)
Negative media and public communication	119 (23.8%)
Religion, culture barriers	43 (8.6%)
Lack of accessibility of services	73 (14.6%)
Lack of trust in authorities	16 (3.2%)
Lack of knowledge and awareness	236 (47.2%)
Poor quality health service experience	30 (6.0%)
Costs associated with vaccination	42 (8.4%)
Knowledge/attitudes of healthcare professionals	36 (7.2%)
Don’t know	70 (14.0%)
Other	135 (27.0%)

Other:

Travelling parents	21 (4.2%)
Irresponsible parents and forget the dates of next dose	21 (4.2%)
Divorced parents	17 (3.4%)
COVID 19 conditions	12 (2.4%)
Live in remote area	11 (2.2%)
Busy parents	9 (1.8%)
Have no ID	9 (1.8%)
Irresponsible parents	8 (1.6%)
Fear from the vaccine	7 (1.4%)
Sick baby	6 (1.2%)
Parents don't know the importance of the vaccine	5 (1.0%)
Family problems	5 (1.0%)

Transportation difficulty	3 (0.6%)
Wrong information on social media	2 (0.4%)
Allergy of the patient	1 (0.2%)

What could your health facility do to improve its immunization coverage:

Sharing data on diseases that can be prevented by vaccination	382 (76.4%)
Sharing information on safety and risk	325 (65.0%)
Explaining why vaccines are recommended and when	406 (81.2%)
Building trust in national decision-making processes	203 (40.6%)
Offer the time, space, and the environment for caregivers to digest information and ask questions	117 (23.4%)
Other	26 (5.2%)
Awareness campaigns and advertising	2 (0.4%)
Communications	3 (0.6%)
Community education	1 (0.2%)
Keep in touch with the mothers	2 (0.4%)
Media campaigns	1 (0.2%)
Reminder for parents for vaccination time	9 (1.8%)
Social campaigns	1 (0.2%)
Social media	1 (0.2%)
Sponsors on social media	1 (0.2%)
Vaccination clinic	1 (0.2%)
We have target	1 (0.2%)
We have weekly data sending to the MOH	3 (0.6%)

To what extent do you agree with the following statements related to your day to day work:

**I have too many responsibilities:**	
Completely disagree	10 (2.0%)
Somewhat disagree	9 (1.8%)

Neither agree nor disagree	21 (4.2%)
Somewhat agree	139 (27.8%)
Completely agree	320 (64.0%)
Don’t know	1 (0.2%)
**I have to attend to too many mothers and children everyday:**	
Completely disagree	59 (11.8%)
Somewhat disagree	23 (4.6%)
Neither agree nor disagree	33 (6.6%)
Somewhat agree	161 (32.2%)
Completely agree	173 (34.6%)
Don't know	51 (10.2%)
**I have far too much to do to be able to explain everything carefully to caregivers:**	
Completely disagree	68 (13.6%)
Somewhat disagree	57 (11.4%)
Neither agree nor disagree	63 (12.6%)
Somewhat agree	160 (32.0%)
Completely agree	141 (28.2%)
Don't know	11 (2.2%)
**I have to spend too much time writing in registers and forms:**	
Completely disagree	78 (15.6%)
Somewhat disagree	77 (15.4%)
Neither agree nor disagree	91 (18.2%)
Somewhat agree	144 (28.8%)
Completely agree	108 (21.6%)
Don’t know	2 (0.4%)

I have to deal with too many caregivers who don't act responsibly for example not showing up for appointments losing their children health cards and not following instructions:	
Completely disagree	76 (15.2%)
Somewhat disagree	70 (14.0%)
Neither agree nor disagree	82 (16.4%)
Somewhat agree	128 (25.6%)
Completely agree	91 (18.2%)
Don't know	53 (10.6%)

I need more training and opportunities for professional advancement:	
Completely disagree	175 (35.0%)
Somewhat disagree	58 (11.6%)
Neither agree nor disagree	58 (11.6%)
Somewhat agree	138 (27.6%)
Completely agree	66 (13.2%)
Don’t know	5 (1.0%)

I need more supervision that is not just criticism but to help me do my job better:	
Completely disagree	216 (43.2%)
Somewhat disagree	110 (22.0%)
Neither agree nor disagree	90 (18.0%)
Somewhat agree	43 (8.6%)
Completely agree	38 (7.6%)
Don’t know	3 (0.6%)

I need more vaccine so I can open a vial for one or a few children as I'm supposed to do:	
Completely disagree	115 (23.0%)
Somewhat disagree	69 (13.8%)
Neither agree nor disagree	122 (24.4%)

Somewhat agree	64 (12.8%)
Completely agree	49 (9.8%)
Don't know	81 (16.2%)

I am not adequately supported and want assurances that the Ministry of Health will defend me against undeserved criticism for example if a child gets sick after vaccination:

Completely disagree	145 (29.0%)
Somewhat disagree	71 (14.2%)
Neither agree nor disagree	94 (18.8%)
Somewhat agree	57 (11.4%)
Completely agree	72 (14.4%)
Don't know	61 (12.2%)

Vaccinating mildly or moderately sick or underweight children may lead to the health worked being blamed by the parents and or the health system if the child's condition worsens:

Completely disagree	113 (22.6%)
Somewhat disagree	58 (11.6%)
Neither agree nor disagree	77 (15.4%)
Somewhat agree	86 (17.2%)
Completely agree	84 (16.8%)
Don't know	82 (16.4%)

Opening a 10 dose or 20 dose vials for one or two children or vaccinating a child from another district or sub district will waste vaccine and lead to stock outs:

Completely disagree	77 (15.4%)
Somewhat disagree	23 (4.6%)
Neither agree nor disagree	66 (13.2%)
Somewhat agree	88 (17.6%)
Completely agree	166 (33.2%)
Don’t know	80 (16.0%)

**Giving multiple vaccinations on the same visit may lead to worse side effects which will upset parents:**	
Completely disagree	166 (33.2%)
Somewhat disagree	75 (15.0%)
Neither agree nor disagree	93 (18.6%)
Somewhat agree	68 (13.6%)
Completely agree	37 (7.4%)
Don't know	61 (12.2%)
**What Health Authorities may do to help improve immunization coverage of facilities**	
Awareness campaigns and information about the latest vaccination studies	204 (40.8%)
Social media	88 (17.6%)
Parents Education about vaccines	49 (9.8%)
Send messages to parents with the next appointments	48 (9.6%)
Availability of the vaccines	46 (9.2%)
Stuff availability	28 (5.6%)
Stuff education	12 (2.4%)
Posters	4 (0.8%)
Brochures	3 (0.6%)

Table S2. Exit interviews with caregivers.

**Type of Vaccination Site: (n=119)**	
Hospital	0 (0.0%)
Primary Healthcare center	119 (100%)
**Sector: (n=119)**	
Government facility	119 (100%)
Private facility	0 (0.0%)
Other	0 (0.0%)

**Are you willing to answer a few questions?**	
Yes	119 (100%)
No	0 (0.0%)
**Why did you come here today?**	
Child's vaccination	119 (100%)
Other	0 (0.0%)
**Did the child receive one or more vaccinations today?**	
Yes	119 (100%)
No	0 (0.0%)
**Why did you come?**	
Scheduled date for vaccination	119 (100%)
Not apply	0 (0.0%)
Nothing	0 (0.0%)
**Q1 - How was your waiting experience today?**	
Bad	0 (0.0%)
Fair	1 (0.84%)
Good	1 (0.84%)
Very Good	41 (34.5%)
Excellent	76 (63.9%)
**Q1 - How was your waiting experience today?**	
B2B	1 (0.84%)
Neutral	1 (0.84%)
T2B	117 (98.3%)
**Q2 - How long did you wait? (Minutes)**	
Minimum to maximum	3 – 60 Minutes
Mean ± SD	11.28 ± 6.31

Median (IQR)	10 (9.0 – 15.0)
**Q3 - What vaccinations did your child receive today?**	
Know	73 (61.3%)
Do not know	46 (38.7%)
**All vaccinations that the child received: (n=73)**	
DPT vaccine	47 (39.5%)
Poliomyelitis	34 (28.6%)
Haemophilus influenza	32 (26.9%)
Hepatic inflammation	28 (23.5%)
Measles	18 (15.1%)
Meningitis	16 (13.4%)
Meningococcal vaccine	13 (10.9%)
Chickenpox	8 (6.7%)
Rotavirus vaccine	7 (5.9%)
Oral Poliomyelitis vaccine	3 (2.5%)
Tuberculosis	2 (1.7%)
Bacterial infection	1 (0.84%)
Mumps	1 (0.84%)
Streptococcus bacteria	0 (0.0%)
**Q4 - Can I please look at your child's vaccination card for a moment to see if what you recall agrees with what the health worker wrote?**	
No	4 (3.3%)
Yes	115 (96.7%)
**What the health worker wrote (or did not write) and on agreement with caregiver's recall: (n=115)**	
The seal	60 (50.4%)
Signature of the health worker & Stamp	47 (39.5%)
Next dose date	59 (49.6%)

DPT bacterial vaccine	1 (0.84%)
Haemophilus influenza	1 (0.84%)
Baby's weight	16 (13.4%)
Following up the child's growth	3 (2.5%)
Writing down that the dose was taken as scheduled	7 (5.9%)
Dosage date	7 (5.9%)
The child who was infected with the Coronavirus, can't be vaccinated now, until after three months of recovery	1 (0.84%)
Not apply	1 (0.84%)
Nothing	50 (42.0%)
**Q5 - Did your child receive all of the vaccinations you came for?**	
Yes	118 (99.2%)
No	1 (0.84%)
**Reasons of not receiving vaccinations: (n=1)**	
My child was infected with Coronavirus and must be vaccinated after 3 months	1 (0.84%)
**Q6 - How did the vaccinator treat you today?**	
Bad	0 (0.0%)
Fair	0 (0.0%)
Good	0 (0.0%)
Very Good	30 (25.2%)
Excellent	89 (74.8%)
**Q7 - Do you need to bring back your child for more vaccinations?**	
Yes	101 (84.9%)
No	17 (14.3%)
Not Sure	1 (0.84%)
**Q8: If yes, do you intend to bring your child back then? (n=101)**	
Yes	101 (100%)
No	0 (0.0%)

Not Sure	0 (0.0%)
**Q9 - Did the vaccinator tell you about how your child might feel after the vaccination?**	
Yes	97 (81.5%)
No	22 (18.5%)
**What did they say to do if your child has some discomfort after the vaccination? (Multiple answer)**	
Give antipyretic	65 (54.6%)
Make compresses	21 (17.6%)
Give analgesic.	31 (26.1%)
Give a suppository	10 (8.4%)
Check the temperature frequently	6 (5.0%)
Not apply	3 (2.5%)
Nothing	0 (0.0%)
**Q10 - Did the vaccinator say anything about your child's vaccination card/health booklet?**	
Yes	53 (44.5%)
No	66 (55.5%)
**Q11 - Do you have any questions about vaccination in general or about today's vaccinations?**	
Yes	29 (24.4%)
No	90 (75.6%)
**Did you ask the health worker? (n=29)**	
Yes	29 (100%)
**Q12 - Is there anything else you would like to mention about your vaccination experience today?**	
The nurse is excellent	1 (0.84%)
Nothing	118 (99.2%)
